# Evaluation of internal farm biosecurity measures combined with sow vaccination to prevent influenza A virus infection in groups of due-to-wean pigs

**DOI:** 10.1186/s12917-022-03494-z

**Published:** 2022-11-09

**Authors:** Gustavo Lopez-Moreno, Cameron Schmitt, Taylor Spronk, Marie Culhane, Montserrat Torremorell

**Affiliations:** 1 Department of Veterinary Population Medicine, College of Veterinary Medicine, University of Minnesota, Saint Paul, MN USA; 2 Pipestone Veterinary Services, Pipestone, MN USA

**Keywords:** Influenza A virus, Pigs, Breeding herds, Internal biosecurity, Management practices

## Abstract

**Background:**

Influenza A virus (IAV) is an important respiratory pathogen of pigs that affects pig health, well-being and productivity, has zoonotic potential, and has significant economic impact for producers. The ultimate goal is to maintain herds free from IAV. Due to the probability of IAV introduction into the herds, it is also desirable for herds to have some immunity to the virus. In this study, we evaluated a protocol that combined sow vaccination with the implementation of internal biosecurity practices during the pre-weaning period with the goal to wean IAV negative pigs.

Five IAV positive breeding herds were vaccinated twice, 3 weeks apart with a herd-specific autogenous vaccine. For the subsequent 8 weeks, a biosecurity protocol was maintained, consisting of no pig movements after 3 days of age, no use of nurse sows, workers changing disposable gloves between litters, workers not stepping into farrowing crates, and daily disinfection of tools and materials used to handle pigs.

**Results:**

Following these interventions, four of the five treatment farms had significant reductions in IAV detection (*p* value < 0.05). Three of the farms tested negative at all sampling points post-intervention and one farm had a 21% reduction in IAV positivity.

**Conclusions:**

This study indicates that a protocol that combines sow vaccination and enhanced biosecurity practices may limit IAV transmission among piglets and enable the weaning of groups of pigs free from the virus.

## Background

Influenza is an important respiratory disease of pigs caused by influenza A virus (IAV) [1]. IAV is endemic in pigs and distributed worldwide with an estimated seroprevalence of approximately 70% [2]. Influenza causes coughing, sneezing, dyspnea and fever [2], affects growth and feed conversion [3] and infected pigs may have increased susceptibility to secondary bacterial infections. The economic impact of influenza has not been well quantified but losses of up to $10 USD per pig have been estimated [4]. IAV is also of public health concern as a multi-host pathogen that can be transmitted between animals and people resulting in zoonotic infections of pandemic potential [5].

Vaccination is the centerpiece of efforts to control IAV in pigs [6]. However, influenza control has become more difficult in the last two decades in part due to the increasing genetic diversity of IAV in pig populations. IAVs with gene segments of avian and human origin have become prevalent in North American pigs and have reassorted with endemic swine IAV strains [7]. The genetic diversity present in swine IAV has been augmented by the emergence and reassortment of the H1N1 2009 pandemic virus into pigs [8] and the on-going spillover of human seasonal viruses [9].

In the USA, the predominant model for swine production is to wean pigs from sow herds around 3 weeks of age and transporting them in groups to separate locations for rearing. Pigs are born IAV negative, but in IAV endemically infected herds pigs commonly become infected prior to weaning [10]. The constant birth of susceptible piglets in breeding herds facilitates the perpetuation of IAV in herds [11]. Currently more than 44 genotypes of IAV have been described in North America [12]. Despite the broad genetic diversity of IAV in pigs, influenza vaccines when administered to pregnant sows can help reduce IAV prevalence in pigs at weaning [13, 14]. However, vaccination alone has not been sufficient to fully prevent infection and enable groups of pigs to be weaned negative for IAV [12, 15].

Weaning groups of IAV negative pigs should limit the losses associated with influenza infections during the post-weaning period [4]. Transport of weaned piglets to distant locations contributes to the dissemination of IAV variants into new regions [16]. Furthermore, there are farm management practices that facilitate IAV dissemination within breeding herds including cross-fostering and use of nurse sows [17, 18]. Nurse sows are lactating sows that, when their original litter is weaned, are used to adopt less viable piglets from other litters. The udders of nurse sows may be contaminated with IAV and serve as a source of infection to the newly adopted pigs [19]. Additionally, mechanical transmission of IAV with contaminated fomites has been shown to disseminate IAV experimentally in low and medium biosecurity settings [20]. Lastly, enhanced biosecurity measures during the pre-weaning period consisting of changing gloves when handling piglets, not stepping into crates and limiting the use of nurse sows has resulted in a reduction and delay of IAV infections in piglets during the first 2 weeks of age. However, these practices were not sufficient to fully prevent IAV infections in the piglets [21]. Although, there are few reports in which pigs have been weaned IAV negative from IAV positive sow herds, these reports usually included interventions after acute outbreaks or when a combination of protocols that included herd vaccination, herd closure and internal biosecurity protocols were implemented. However, these reports are scarce and further studies are needed to validate these protocols [22–24].

We hypothesize that in order to break the cycle of IAV infection in piglets, a combination of sow vaccination and internal biosecurity practices during the pre-weaning period are necessary to prevent infection in the pigs and enable weaning of IAV negative groups. Reducing the risk of IAV exposure to piglets and increasing the resistance of the piglets to infection by having pigs with maternally derived antibodies should minimize the risk of piglets from becoming infected. Ultimately, controlling influenza in pigs will result not only in improved animal health and well-being, but also will decrease the risk of infections to people.

## Methods

### Study design

Six IAV positive breeding herds farrow to wean were conveniently selected to participate in this observational study. The farms were located in the US Midwest States, belonged to the same production company and had an average inventory of 3500 sows. Piglets were weaned 2-3 times a week and during the six months prior to the study, there were no vaccinations against IAV in neither the sows nor the piglets. All the pigs in the study were from commercial farms and introduced replacement animals from external sources. Sows were a crossbred of Landrace and Yorkshire breeds. Farms had between 12 and 18 farrowing rooms and sows were moved into the farrowing rooms approximately 2 days before parturition. Piglets were born into the farrowing rooms and reared with the sow until approximately 18-22 days of age. At weaning pigs were transported to a nursery or wean-to-finish facility located in a separate site and were managed according to the company’s standard operational procedures until harvest. After weaning, there was no further sampling of the piglets or the sows. The farms were part of an IAV monthly monitoring program and were selected based on their history of IAV positivity at weaning and their willingness to adhere to study protocol. The study took place during the peak of the influenza season in pigs from November 2020 to March 2021 [25].

Five of the farms were assigned to the treatment group conveniently and one of the farms was used as control. Farms in the treatment group combined the use of whole herd sow and gilt vaccination with implementing an enhanced internal biosecurity protocol. This protocol consisted of no moving pigs between litters after 3 days of age; not using nurse sows to adopt fall behind piglets; workers changing disposable gloves between litters when handling piglets; not stepping into farrowing crates; and conducting daily disinfection of tools and materials used to handle the piglets. Farmworkers were also segregated in blocks in order to avoid workers to handle newborn piglets after handling older piglets. All sows and gilts were vaccinated twice, intra-muscularly approximately 3 weeks apart, with a herd-specific licensed autogenous vaccine. The autogenous vaccine included two strains of the H1 subtype (delta-1-1B.2.2.1 and pdm-vaccine-1A.3.3.2) and one of the H3 subtype (Cluster IVA 3.1990.4.1) and strains had been selected by genetically analyzing the company’s IAV isolates recovered from the company’s farms and selecting the most prevalent. Replacement gilt introduction was modified to take place after a quarantine period of 42 days. In the control farm, there were no changes to the routine management practices and IAV vaccination was not used. This farm served as a control for IAV status changes not due to study protocol.

### Sampling protocol and specimen collection

**Fig. 1:**
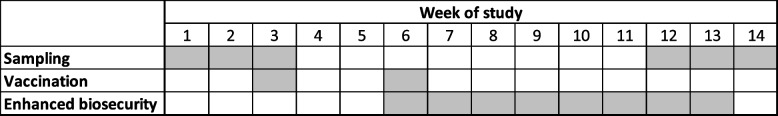
Timeline in weeks of intervention activities and sample collection events. Gray cells indicate when the activities took place

Before implementing the study protocol all farms were sampled for 3 consecutive weeks to assess IAV presence (Fig. 1). Each week, thirty udder skin wipes were collected from due-to-wean lactating sows to detect at least one positive litter when the prevalence was 10% or higher, with a 95% confidence. The sows were randomly selected within the 3-4 farrowing rooms that had due-to-wean piglets. Udder skin sampling consists of using a moistened wipe on the surface of the udder skin of the lactating sow to indirectly sample piglets’ nasal and oral secretions to assess litter IAV status [19]. Environmental samples were also collected from farrowing rooms with piglets of weaning age by placing two 3-ft long aluminum foil pieces in each room on top of farrowing crates and sampling their surface after one hour with a moist sterile wipe [19].

Approximately six weeks after the second dose of vaccine was applied (post treatment sampling), 30 litters of weaning age were sampled for 3 consecutive weeks for a total of 90 litters as mentioned above to assess the IAV positivity after intervention. The 6-week interval between the second vaccination and sampling was selected to ensure that samples were collected from litters in which the sow had two doses of vaccine and had enough time to develop IAV specific antibodies to transfer to her piglets through colostrum.

Sampling wipes consisted of 3 × 3 in. cotton sterile gauzes moistened with 10 ml of DMEM-Dulbecco’s Modified Eagle Medium Gibco™(Grand Island, NY, USA) supplemented with 0.5 ml of Gentamicin Sulfate (BioWhittaker®, Walkersville, MD USA) and 5 ml of antibiotics and antimycotic, Anti-Anti (100x) Gibco™(Grand Island, NY, USA). All samples were collected by study personnel and transported refrigerated using ice packs to the University of Minnesota research laboratories, where samples were aliquotted and stored frozen at − 80 °C until testing.

### Influenza A virus detection assays

RNA was extracted from all samples using the magnetic particle processor procedure (Ambion® MagMAX™AM1836, Viral RNA Isolation Kit; Applied Biosystems, Foster City, CA, USA). Samples were tested individually using a real-time reverse-transcriptase polymerase chain reaction (rRT-PCR) that targets the IAV conserved matrix gene using previously described procedures [26]. Samples with cycle threshold (ct) values below 37 were considered positive.

### Statistical analysis

Data from the rRT-PCR results were consolidated in a spreadsheet (Microsoft Excel, Microsoft Corporation, Redmond, Washington, USA) and organized for analysis. Means, minimum and maximum values for quantitative variables, and frequency counts and percentages for qualitative variables were calculated for descriptive analysis. Differences in IAV positivity before and after the intervention by farm were assessed with a Fisher’s exact test using R statistical software (version 4.1.1) [27].

## Results

### Farm IAV status prior to whole herd sow vaccination and implementation of biosecurity protocols

**Fig. 2:**
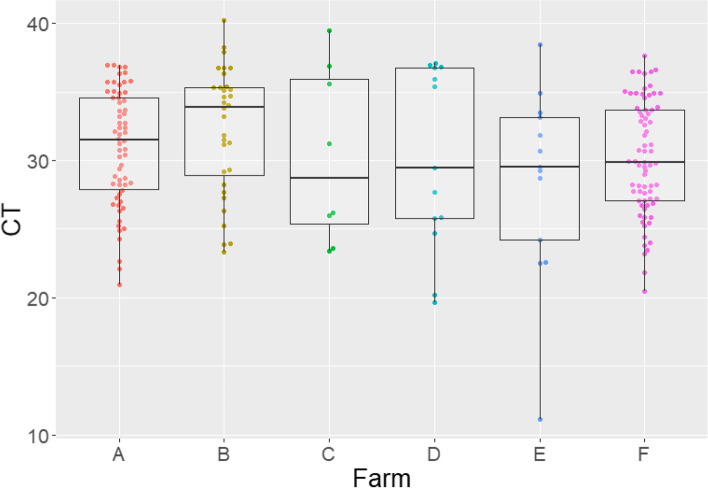
Distributions by farm of influenza A virus positive and suspect cycle threshold (Ct) values obtained by rRT-PCR from udder skin wipes collected prior to whole herd sow vaccination and implementing enhanced biosecurity practices. Boxes depict 50% of the data points and the line represents the median Ct values for the corresponding farm

All farms tested IAV positive in the three samplings done before implementing the internal biosecurity and vaccination protocols, except for farm C that tested IAV positive in 2 of the 3 sampling points. The overall litter IAV positivity rate before protocol implementation ranged from 8 to 81% (Table 1). The cycle threshold results obtained by rRT-PCR ranged from 11 to 40 (Fig. 2).

**Table 1:** Number (percentage) of influenza A virus rRT-PCR positive udder skin wipes by farm and sampling event before whole herd sow vaccination and implementing enhanced biosecurity practices

Experimental group	Farm	Sampling events			
		Week 1 (%)	Week 2 (%)	Week 3 (%)	Total (%)
Treatment	A	28/30^a^ (93.3)	28/30 (93.3)	6/30 (20)	62/90 (68.9)
Treatment	B	3/30 (10)	2/30 (6.7)	26/30 (86.6)	31/90 (34.4)
Treatment	C	2/30 (6.7)	0/30 (0)	5/30 (16.7)	7/90 (7.8)
Treatment	D	5/30 (16.7)	6/30 (20)	2/30 (6.7)	13/90 (14.4)
Treatment	E	4/30 (13.3)	3/30 (10)	5/30 (16.7)	12/90 (13.3)
Control	F	25/30 (83.3)	25/30 (83.3)	23/30 (76.7)	73/90 (81.1)

Udder skin wipes were tested individually. A specimen was considered positive when the rRT-PCR cycle threshold < 37

Sampling events refer to weeks 1, 2 and 3 of the study

^a^Number of positive litters / total number of litters tested (percentage)

Environmental samples from all farms with exception of farm C tested rRT-PCR positive at all three sampling events (Table 2). Overall, the percentage of positive environmental samples ranged between 8 and 81%.

**Table 2:** Number (percentage) of influenza A virus rRT-PCR positive environmental samples by farm and sampling event before whole herd sow vaccination and implementing enhanced biosecurity practices

Experimental group	Farm	Sampling events			
		1 (%)	2 (%)	3 (%)	Total (%)
Treatment	A	6/6^a^ (100)	6/6 (100)	1/4 (25)	13/16 (81.2)
Treatment	B	2/6 (33.3)	3/8 (37.5)	6/6 (100)	11/20 (55)
Treatment	C	0/6 (0)	0/8 (0)	2/10 (20)	2/24 (8.3)
Treatment	D	Not collected	2/6 (33.3)	3/8 (37.5)	5/14 (35.7)
Treatment	E	2/8 (25)	2/10 (20)	1/6 (16.7)	5/24 (20.8)
Control	F	4/6 (67.7)	4/6 (67.7)	6/6 (100)	14/18 (77.7)

Environmental samples were tested individually. A specimen was considered positive when the rRT-PCR cycle threshold was < 37

Sampling events refer to weeks 1, 2 and 3 of the study

^a^Number of positive samples / total number of samples tested (percentage)

### Farm IAV status after whole herd sow vaccination and implementation of biosecurity protocols

Significant reductions in the IAV positivity rate of udder wipes were observed on farm B (*p* value = 0.002), farm C (*p* value = 0.01), farm D (*p* value = < 0.0002) and farm E (*p* value = 0.0003) (Table 5). On farms C, D and E, all post-intervention udder wipe and environmental samples tested IAV negative (Tables 3 and 4). In contrast, IAV positivity rate in farm A remained high in both set of samples. Farm F which was the control farm continued to test IAV positive at all three sampling events.

**Table 3:** Number (percentage) of influenza A virus rRT-PCR positive litters by farm and sampling event after whole herd sow vaccination and implementing enhanced biosecurity protocols

Experimental group	Farm	Sampling events			
		Week 12 (%)	Week 13 (%)	Week 14 (%)	Total (%)
Treatment	A	15/30^a^ (50)	16/30 (53.3)	25/30 (83.3)	56/90 (62.2)
Treatment	B	11/30 (36.7)	0/30 (0)	1/30 (3.3)	12/90 (13.3)
Treatment	C	0/30 (0)	0/30 (0)	0/30 (0)	0/90 (0)
Treatment	D	0/30 (0)	0/30 (0)	0/30 (0)	0/90 (0)
Treatment	E	0/30 (0)	0/30 (0)	0/30 (0)	0/90 (0)
Control	F	21/30 (70)	20/30 (66.7)	30/30 (100)	71/90 (78.9)

Udder skin wipes were tested individually. A specimen was considered positive when the rRT-PCR cycle threshold was < 37

Sampling events refer to weeks 12, 13 and 14 of the study

^a^Number of positive litters / total number of litters tested (percentage)

**Table 4:** Number (percentage) of influenza A virus rRT-PCR positive environmental samples by farm and sampling point event after whole herd sow vaccination and implementing enhanced biosecurity protocols

Experimental group	Farm	Sampling events			
		Week 12 (%)	Week 13 (%)	Week 14 (%)	Total (%)
Treatment	A	10/10^a^ (100)	6/14 (42.9)	6/6 (100)	22/30 (73.3)
Treatment	B	6/6 (100)	0/10 (0)	1/6 (17)	7/22 (31.8)
Treatment	C	0/8 (0)	0/8 (0)	0/8 (0)	0/24 (0)
Treatment	D	0/8 (0)	0/8 (0)	0/8 (0)	0/24 (0)
Treatment	E	0/6 (0)	0/6 (0)	0/4 (0)	0/16 (0)
Control	F	6/6 (100)	3/3 (100)	4/4 (100)	13/13 (100)

Environmental samples were tested individually. A specimen was considered positive when the rRT-PCR cycle threshold < 37

Sampling events refer to 12, 13 and 14 weeks after biosecurity protocol was initiated

^a^Number of positive samples / total number of samples tested (percentage)

Environmental sampling results were similar to those obtained from the udder wipes, with all post-intervention samples testing negative on farms C, D and E. IAV positivity rate in farm B environmental samples was numerically lower (32% vs. 55%) but the difference was not statistically significant. Farm A and farm F tested IAV positive consistently to the end of the study (Table 4).

Overall, there was a significant reduction in the proportion of IAV positives in all the farms on which the intervention was applied with the exception of Farm A (Table 5).

**Table 5:** Number (percentage) of influenza A virus rRT-PCR positive litters before and after interventions measured by udder skin wipes by farm. Statistical significance was measured between pre-intervention and post-intervention positive proportions

Experimental group	Farm	Pre-intervention IAV positivity (%)	Post-intervention positivity (%)	*P* value^^^
Control	F	73/90^a^ (81.1)	71/90 (78.9)	0.85
Treatment	A	62/90 (68.9)	56/90 (62.2)	0.43
Treatment	B	31/90 (34.4)	12/90 (13.3)	0.002
Treatment	C	7/90 (7.7)	0/90 (0)	0.01
Treatment	D	13/90 (14.4)	0/90 (0)	0.0002
Treatment	E	12/90 (13.3)	0/90 (0)	0.0003
Total treatment^b^	–	125/450 (27.8)	68/450 (15.1)	< 0.0001

^a^Number of positive samples / total number of samples tested (percentage)

^b^Total values were summarized using farms assigned to the treatment group

^*P* values were obtained using a Fisher’s Exact test

## Discussion

The most vulnerable phases in the life of growing pigs are immediately following birth and in the period following weaning. Maintaining good health and appetite in weaned pigs is an important determinant of their future and growth rate, and avoiding infectious disease challenges during this phase is critical [28]. Weaning groups of pigs that are free from IAV infection is therefore advantageous to swine producers because of the enhanced health and productivity benefits, but also reduces the spread of IAV to other locations and zoonotic risks to the public. In this study, we evaluated the impact of a combination of whole herd sow vaccination with enhanced internal biosecurity practices during the pre-weaning period, on the positivity rates of IAV at the time of weaning. Individually, these approaches have been shown to delay [21] or reduce IAV infections at weaning [13] but have typically not resulted in elimination of the virus. In the current study, the strategic combination of both interventions (vaccination and enhanced internal biosecurity) showed considerable promise as a means to substantially reduce and potentially eliminate IAV infections in groups of weaned pigs. However, this approach was not universally successful, and further research will be required to understand determinants of success, refinement to the approach and how to achieve effective compliance on farms.

Successful reduction of IAV risk may be related to baseline IAV detection in the target populations. In our study, the three farms with the lowest baseline of IAV positivity rate (8 and 14%) were those that uniformly tested IAV negative after completion of the protocol. In contrast, the two farms with higher IAV positivity rates (34 and 69%) tested positive after protocol implementation. Farm B had significantly lower positivity rates, but there was still evidence of virus persistence in the population at the final sampling event. In contrast, positivity rate in farm A was unchanged (69% vs. 62%). Higher baseline positivity rates may lead to higher environmental contamination and risk of mechanical dissemination of IAV to susceptible pigs. Also, the follow up period of this study was only 3 weeks, and more time may be necessary to achieve reduction and/or elimination of IAV from populations with high IAV positivity rates.

The reduction and/or apparent elimination of IAV from 4 of the 5 farms was likely the result of the direct implementation of the interventions rather than changes in IAV seasonality. The study took place during the fall and winter months which is when it is considered “flu season” in the North American Midwest [25], and the study was completed over a relatively short time period. However, long-term controlled studies are needed to better assess the efficacy of the approach.

The vaccines used in this study were customized autogenous vaccines targeting selected IAV strains that were predominant in the production company’s farms prior to the pre-study period. Due to the antigenic variability of IAV viruses, and the limited and variable cross-protection among them, incorporating existent variants into vaccines for sows should maximize the transfer of maternal immunity and limit virus replication [14, 29]. Although we cannot ensure that all the circulating strains in the farms were represented in the vaccines, the multivalent strain combination likely played a role in inducing maternally derived immunity. However, we anticipate that success of this approach is likely to be influenced by the correspondence between vaccine strains and those actually circulating in herds. Nevertheless, even when vaccine components are not completely homologous with circulating IAV, vaccination may still provide some beneficial effect [13].

The reasons why no material reduction of IAV positivity rate was seen at farm A have not been elucidated. The diagnostics performed as part of the investigation did not reveal additional IAV strains distinct from the vaccine viruses. Also, it is likely that compliance in implementing the biosecurity protocols varied. The protocols used contained some standard management practices (e.g., cross-fostering and use of nurse sows) that are used to enhance the nutrition and well-being of the suckling pigs. Issues of compliance are therefore to be expected, however we were unable to assess compliance by workers on the farms. Lastly, given the significant infection levels and environmental contamination, it is possible that other means of indirect transmission (i.e., air) may have played a role in maintaining infections in the piglets.

The timing and interval between the implementation of vaccination and enhanced internal biosecurity measures was chosen in order to allow sufficient time for sows to develop an immune response to vaccination that would provide maternally derived immunity in their offspring. Implementation of the strict biosecurity protocol was restricted to 8 weeks to minimize potential piglet losses due to the constrains on normal management practices. It is likely that certain farms may benefit from implementing protocols over a longer period, however a cost benefit analysis should be performed. Alternatively, the protocol could be implemented in a phased manner in which the strict biosecurity protocol would not be initiated until a threshold of low IAV positivity was achieved. Additional studies are needed to further evaluate modifications to the protocol on both IAV positivity rates at weaning and productivity parameters in the pre and post-weaning periods. However, given the economic burden of IAV and the potential role that pigs have in generating new IAV strains with zoonotic potential, eliminating IAV from suckling piglets in breeding herds should out-weigh any temporary economic losses due to protocol implementation.

A point of consideration when evaluating protocols for disease elimination is the duration of sampling after the protocol has been implemented. Three weeks were chosen for logistic reasons and because in the course of 3 weeks all the farrowing rooms at the facilities would be included. Furthermore, we anticipated a rapid impact of the protocol given the knowledge on IAV transmission and incubation of IAV in pigs [2, 30]. However, the collaborating production company continued monitoring IAV status of the pigs monthly after the study was terminated, by collecting nasal swabs of piglets of weaning age and communicated that negative results in all farms except for farm A continued for at least 6 months after the implementation of the protocol. Another point to consider when evaluating the effectiveness of the interventions is sampling of the pig population once piglets have been weaned. This would have been ideal but access to samples from weaned pigs was not an option in this study. Nevertheless, future studies should include the evaluation of interventions on breeding herds not only in piglets prior to weaning but also during the post-weaning period.

Another point of consideration is that we focused our efforts in sampling and implementing strategies impacting the piglet population. We did not pursue sampling of other populations such as gilts or sows. Replacement animals IAV status is considered a risk factor for herds weaning IAV positive piglets [31], therefore incorporating a protocol for replacement animal introduction should be essential for the long-term IAV exclusion from breeding herds.

The results from the environmental samples were generally in agreement with those from sow udder skin samples, and validated the sensitivity of this sampling method to detect IAV at the farm level, even when the IAV positivity rates at the litter level were low. Environmental sampling may be a more cost-effective option for the long-term screening of breeding herds for IAV.

## Conclusion

In summary, this study indicates evidence that combined sow vaccination and enhanced internal biosecurity has considerable promise for reducing IAV infection in piglets and even eliminating IAV in groups of due-to-wean pigs. Application of this approach should aid to decrease the burden of IAV infections in swine populations and should help limit risk of IAV of swine origin in people. However, the low number of farms, the short follow-up period and the usage of licensed custom IAV vaccines are all factors that have to be considered when interpreting the results obtained as part of this study.

## Data Availability

The datasets used and/or analyzed during the current study are available from the corresponding author on reasonable request.

## Abbreviations

IAV: Influenza A virus
rRT-PCR: real-time reverse-transcriptase polymerase chain reaction
RNA: Ribonucleic acid
Ct: Cycle threshold
